# α_1D_-Adrenoceptors are responsible for the high sensitivity and the slow time-course of noradrenaline-mediated contraction in conductance arteries

**DOI:** 10.1002/prp2.1

**Published:** 2013-08-28

**Authors:** Nicla Flacco, Jaime Parés, Eva Serna, Vanessa Segura, Diana Vicente, Miguel Pérez-Aso, María Antonia Noguera, María Dolores Ivorra, John C McGrath, Pilar D'Ocon

**Affiliations:** 1 Departamento de Farmacología, Facultad de Farmacia, Universitat de ValènciaValencia, Spain; 2 Autonomic Physiology Unit, School of Life Sciences, College of Medical, Veterinary and Life Sciences, University of GlasgowGlasgow, U.K

**Keywords:** α_1A_-adrenoceptors, conductance and resistance vessels, contraction time-course

## Abstract

The objective of this study was to determine whether the different time-course characteristics of α_1_-adrenoceptor-mediated contraction in arteries can be related to the subtypes involved. Contractile responses to noradrenaline (NA) were compared with inositol phosphate accumulation and extracellular signal-regulated kinase (ERK)1/2 phosphorylation after α_1_-agonist stimuli in the same vessels in the presence or absence of α_1_-antagonists in rat or in α_1_-subtype knockout (KO) mice. Aorta, where α_1D_-AR is the main functional subtype, had higher sensitivity to NA (in respect of inositol phosphate [IP], pERK1/2, and contractile response) than tail artery, where the α_1A_-adrenoceptor subtype is predominant. Furthermore, the contraction in aorta exhibited a slower decay after agonist removal and this was consistent in all strains harboring α_1D_-adrenoceptors (from rat, α_1B_-KO, and wild-type [WT] mice) but was not observed in the absence of the α_1D_-adrenoceptor signal (α_1D_-adrenoceptor blocked rat aorta or aorta from α_1D_-KO). IP formation paralleled α_1_-adrenoceptor-mediated contraction (agonist present or postagonist) in aorta and tail artery. High sensitivity to agonist and persistence of response after agonist removal is a property of α_1D_-adrenoceptors. Therefore, the preponderance of this subtype in noninnervated conductance arteries such as aorta allows responsiveness to circulating catecholamines and prevents abrupt changes in vessel caliber when the stimulus fluctuates. Conversely, in innervated distributing arteries, high local concentrations of NA are required to activate α_1A_-adrenoceptors for a response that is rapid but short lived allowing fine adjustment of the contractile tone by perivascular sympathetic nerves.

## Introduction

The α_1_-adrenoceptors (ARs) are responsible for the contractile response to catecholamines in blood vessels and, classically, three different subtypes have been characterized, α_1A_, α_1B_, and α_1D_-ARs. The presence of mRNA or receptor protein is not well correlated with contractile function; in several examples, mRNA and protein for all three α_1_-AR subtypes are expressed in a vessel, yet pharmacological analysis shows that a single subtype is mainly responsible for mediating contraction. There is, however, some correlation between the subtype involved in mediating vascular contraction and the type of vessel. For example, α_1A_-ARs mediate contraction of well-innervated distributing arteries such as renal (Hrometz et al. 1999), tail (Lachnit et al. 1997; Tanaka et al. 2004), and distal mesenteric and resistance arteries such as small mesenteric branches (Philipp and Hein 2004; Martí et al. 2005; Methven et al. 2009a). On the other hand the α_1D_-AR has been shown to regulate the contraction of poorly innervated conductance arteries such as the aorta, femoral, iliac, carotid, pulmonary, and superior mesenteric artery (Piascik et al. 1995; Hussain and Marshall 1997; Rudner et al. 1999; Gisbert et al. 2000; Arévalo-León et al. 2003; Martí et al. 2005; Methven et al. 2009b) and there is only limited direct evidence that the α_1B_-AR is a mediator of contractile function in blood vessels (Cavalli et al. 1997; Daly et al. 2002; Tanoue et al. 2003; Cotecchia 2010; Docherty 2010). Thus, vascular α_1_-AR subtypes may correlate with the different functions of smooth muscle in these different vascular types, that is, compliance of large arteries (α_1D_) and redistribution of blood flow between different organ systems (α_1A_) as we have previously discussed (Daly et al. 2002; Ziani et al. 2002).

In general, α_1_-ARs manifest different sensitivity to agonists, the α_1D_-subtype being the most sensitive (Theroux et al. 1996; Taguchi et al. 1998; Gisbert et al. 2000; Piascik and Perez 2001; Daly et al. 2002). Once activated, the three α_1_-AR subtypes interact with the Gq protein but can also activate a variety of other signaling pathways such as Gi and Go proteins or mitogen-activated protein kinases (MAPKs) (Hawrylyshyn et al. 2004; Hein and Michel 2007; Cotecchia 2010) that are less well explored in native tissues. Nevertheless, there are marked differences in the ability of each subtype to generate intracellular second messengers (García-Sainz et al. 1999b; Zhong and Minneman 1999; Keffel et al. 2000; Piascik and Perez 2001). The α_1A_-AR is most efficiently coupled to inositol phosphate production, increases in cytosolic calcium concentrations, and MAPKs pathway, whereas the α_1D_-AR is poorly coupled to intracellular signaling cascades (Schwinn et al. 1991; Theroux et al. 1996; Taguchi et al. 1998; Zhong and Minneman 1999; García-Sainz and Villalobos-Molina 2004; Hein and Michel 2007; García-Cazarín et al. 2008). This points to the possibility that potential differences in their efficacy result in different functional outcomes for each α_1_-AR subtype.

There are more observations that add complexity to this scenario. As previous results obtained by our research group indicate, native α_1D_-AR remains active after removing the agonist (Noguera and D'Ocon 1993; Noguera et al. 1996; Gisbert et al. 2000, 2002, 2003b; Ziani et al. 2002) in vessels where this subtype play a functional role. They act as “*constitutively active*” receptors which maintain an increased vascular tone for some time after the adrenoceptor-mediated stimulus is removed (Ziani et al. 2002). This constitutive activity of α_1D_-ARs has also been found in stably transfected rat fibroblasts and HEK293 cells where a α_1D_-mediated pERK1/2 signal was observed in the absence of an adrenoceptor-mediated stimulus (García-Sainz and Torres-Padilla 1999a; McCune et al. 2000; Chalothorn et al. 2002; Pérez-Aso et al. 2013).

We propose that the characteristic behavior of the α_1D_-subtype: higher sensitivity, sustained activity after removal of the agonist, and its presence in the poorly innervated conductance vessels, could determine a distinctive time-course of the adrenoceptor-mediated contraction in these vessels, and permit them to respond to the circulating levels of catecholamines (rarely above 10 nmol/L) (Goldstein et al. 2003).

In the present work, we confirm this hypothesis by analyzing the characteristics of the response elicited by α_1_-ARs in two different vessels, aorta, a territory where the α_1D_-AR subtype plays the main functional role, and tail artery as a vessel where the α_1A_-AR subtype is the main one responsible of the adrenoceptor-mediated contractile response. Involvement of subtypes was manipulated by the use of selective antagonists in the rat and subtype knockouts in the mouse (α_1B_-KO, α_1D_-KO, and α_1B/D_-KO). Signaling pathways were investigated alongside contractility studies by analyzing inositol phosphate accumulation and extracellular signal-regulated kinase (ERK)1/2 phosphorylation after adrenoceptor stimulus in the same vessels.

## Materials and Methods

### Animals

Thoracic aorta and tail artery were obtained as previously described (Gisbert et al. 2000) from male Wistar rats (200–250 g) from colonies of wild-type (WT) and α_1D_-KO mice, kindly supplied by Professor Gozoh Tsujimoto (Department of Molecular Cell Pharmacology, National Research Institute for Child Health and Development, Tokyo), α_1B_-KO mice, kindly supplied by Professor Susanna Cotecchia (Départment de Pharmacologie et de Toxicologie, Université de Lausanne, Switzerland), and α_1B/D_-KO mice generated by crossing these α_1D_-KO and α_1B_-KO strains at University of Glasgow (see Methven et al. 2009a). All protocols complied with European Community guidelines for experimental animals and were approved by the Ethics Committee of the University of Valencia.

### Functional studies

Rings obtained from rat vessels and mouse aorta, were denuded of endothelium by gentle rubbing and suspended in an organ bath. Tension was recorded isometrically according to the protocol previously described (Martí et al. 2005). Arterial rings from the mouse tail were mounted on an isometric wire myograph (J.P. Trading, Aarhus, Denmark) according to the procedure previously described (Martinez-Rivelles et al. 2012). All vessels were maintained in Krebs buffer, at 37°C and gassed with 95% O_2_ and 5% CO_2_.

An initial load of 9.81 mN was applied to each preparation and maintained throughout a 75–90 minutes equilibration period. The rings were stimulated with noradrenaline (NA) (10 μmol/L in tail artery or 1 μmol/L in aorta) which produced a maximal contraction. The lack (<10%) of a relaxant response to acetylcholine (100 μmol/L) in these precontracted preparations indicated the absence of a functional endothelium. After 30 minutes washout, contractile responses to NA were elicited according to different experimental procedures:

1. *Concentration–response curves (CRC) to NA*. This experimental procedure was performed in each vessel by addition of cumulative concentrations of NA (0.0001–10 μmol/L) until a maximal response was obtained. From these curves, pD_2_ and Emax were calculated using a nonlinear regression plot (Graph Pad Software; San Diego, CA).
2. *Sustained contractile response to NA*. The experimental procedure was performed according to previous studies (Noguera and D'Ocon 1993; Gisbert et al. 2000; Ziani et al. 2002). A maximal contractile response to NA (1 μmol/L in aorta, 10 μmol/L in tail artery) was obtained in Ca^2+^-containing medium; this concentration was maintained until a stable tone was reached and then washed. In some experiments, BMY 7378 or 5-methylurapidil were added 15 minutes prior to NA addition. The washing procedure was carried out with a total replacement of the bathing solution by three repeated washes within the first 30 seconds and by two other repeated washes every 5 minutes in all cases. The tone was measured at different times during the development of the contractile response and after washing until total recovery of the basal tone. The results were expressed as percentages of maximal contractile responses.
3. *Increase in vascular tone after removal of the agonist*. Vessels were incubated in a Ca^2+^-free solution (containing 0.1 mmol/L ethylenediaminetetraacetic acid, EDTA) for 20 minutes, which led to a small loss in tension (<10–15%); then, vessels were exposed to NA (1 μmol/L in aorta, 10 μmol/L in tail artery) twice, 10 minutes each time, the tissues being carefully washed between the two exposures, following the same procedure described above. A spontaneous increase in vascular tone was observed when the Ca^2+^-free solution was substituted by a Ca^2+^-containing medium. The effects of prazosin, 5-methylurapidil, and BMY 7378 were assessed on this spontaneous increase in tone. One micromolar of each antagonist was added during incubation in Ca^2+^-free medium, 10 minutes before addition of Ca^2+^-containing solution and was maintained during the Ca^2+^-loading period. Contraction was expressed in mN.

### Accumulation of [^3^H]-inositol phosphates

The determination of the accumulation of inositol phosphates (IPs) has been previously described (Gisbert et al. 2003b). Briefly, rat tail arteries or thoracic aortas were cut into rings, pooled, and submitted to different experimental procedures:

1. Incubation for 30 minutes with increasing concentrations of NA (0.01 μmol/L–0.1 mmol/L) in the presence of LiCl (10 mmol/L) in order to inhibit the metabolism of inositol monophosphates.
2. Incubation for 30 minutes with 1 μmol/L of prazosin, 5-methylurapidil, or BMY 7378, in the presence of LiCl (10 mmol/L) and in presence or absence of NA (1 μmol/L in aorta, 10 μmol/L in tail artery).
3. Incubation for 30 minutes with NA (1 μmol/L in aorta, 10 μmol/L in tail artery) in Ca^2+^-free medium in the absence of LiCl to avoid accumulation of inositol phosphates, followed by removal of the agonist by careful washing, and incubation for 30 minutes in Ca^2+^-containing medium in the presence of LiCl (10 mmol/L).

At the end of the functional experiments all vessels were immediately frozen and processed as previously published (Monto et al. 2012) to obtain total proteins.

Accumulation of [^3^H]-IPs was routinely calculated as dpm of total [^3^H]-inositol labeled lipids/μg of protein in each individual sample. CRC for NA-induced [^3^H]-IPs accumulation were fitted by nonlinear regression plot (Graph Pad Software; San Diego, CA) and the pEC_50_ and Emax values were obtained.

### Determination by immunoblotting of NA-mediated ERK1/2 activation

Rings of rat aorta or tail artery were loaded in tubes containing 5 mL of Krebs solution gassed with 95% O_2_ and 5% CO_2_, at 37°C. After 30 minutes of stabilization in Ca^2+^-containing medium, selective antagonists were added when indicated, and maintained for 15 minutes. Aorta and tail artery segments were then stimulated or not with NA for 5 minutes and then the tissues were immediately frozen by liquid N_2_ immersion.

Protein extracts (50 μg) were loaded onto 10% Sodium dodecyl sulphate-Polyacrylamide gels, and electrophoresed proteins were transferred to polyvinyldiene fluoride (PVDF) membranes 2 hours at 375 mA, using a liquid Mini Trans-Blot® Electrophoretic Transfer Cell system (Bio-Rad Laboratories, Inc., S. A. Madrid, Spain). Membranes were blocked in albumin from bovine serum 3% in phosphate-buffered saline (PBS) containing 0.1% Tween 20 for 1 hour at room temperature with gentle agitation. Membranes were incubated overnight at 4°C with anti-phospho-p42/44 ERK MAPK (Thr202/Thr204) and anti-p42/44 ERK MAPK (1:500; Cell Signaling Technology, Beverly, MA). Membranes were then washed three times with PBS with 0.1% Tween 20, incubated with anti-rabbit immunoglobulin G horseradish peroxidase-linked whole antibody (1:2500; GE Healthcare, Buckinghamshire, U.K.) for 45 minutes at room temperature and washed extensively with phosphate buffered saline with tween before chemiluminescent detection was performed using the the ECL™ Prime Western Blotting Detection Reagents (GE Healthcare, Buckinghamshire, U.K.). The image was captured with the AutoChemi System (Ultra-Violet Products Bioimaging Systems, Cambridge, U.K.) and band intensity was measured using LabWorks 4.6 Image acquisition and Analysis (Ultra-Violet Products Bioimaging Systems, Cambridge, U.K.).

### Drugs and solutions

The following drugs were obtained from SIGMA (St. Louis, MO): (-)-NA, prazosin, BMY 7378 (8-[2-[4-(2-Methoxyphenyl)-1-piperazynil]-8-azaspiro [4,5]decane-7,9-dione dihydrochloride) and 5-methylurapidil. Other reagents were of analytical grade. All compounds were dissolved in distilled water. The composition of Krebs solution was (mmol/L) NaCl 118, KCl 4.75, CaCl_2_ 1.8, MgCl_2_ 1.2, KH_2_PO_4_ 1.2, NaHCO_3_ 25, and glucose 11. Ca^2+^-free solution had the same composition except that CaCl_2_ was omitted and EDTA (0.1 mmol/L) was added. The terminology for receptors employed is as recommended in Alexander et al. (2011).

## Results

### NA exhibits higher potency but lower efficacy in aorta than in tail artery

**Figure 1:**
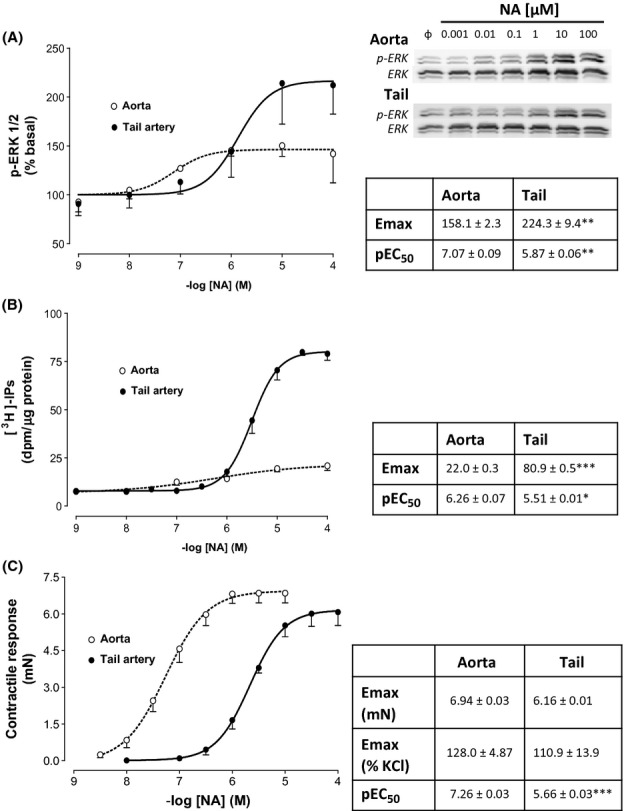
Concentration–response curves of noradrenaline (NA) on (A) p-ERK signaling pathway determined by immunoblotting. A representative immunoblot was also included, (B) inositol phosphates accumulation (IPs), (C) vascular tone expressed as force units (mN). Experiments were performed in aorta (white circles) and tail artery (black circles) from rat. Emax and pEC_50_ of the concentration–response curves were included in each case. Values are represented as mean ± SEM of n = 3–6 experiments. Statistical signification was calculated by Student's *t* test. *P < 0.05, ***P < 0.001.

NA elicited a concentration-dependent contraction, ERK 1/2 phosphorylation, and [^3^H]-IPs accumulation in both rat aorta and tail artery (Fig. 1). The potency (pEC_50_) of NA was higher in aorta than in tail artery, for all three measures; conversely, this increase in potency was accompanied by an apparently lower efficacy in IPs formation and ERK1/2 phosphorylation in aorta when normalized for protein content (Fig. 1A and B). It is not practicable to compare contractile efficacy between different vessels.

**Figure 2:**
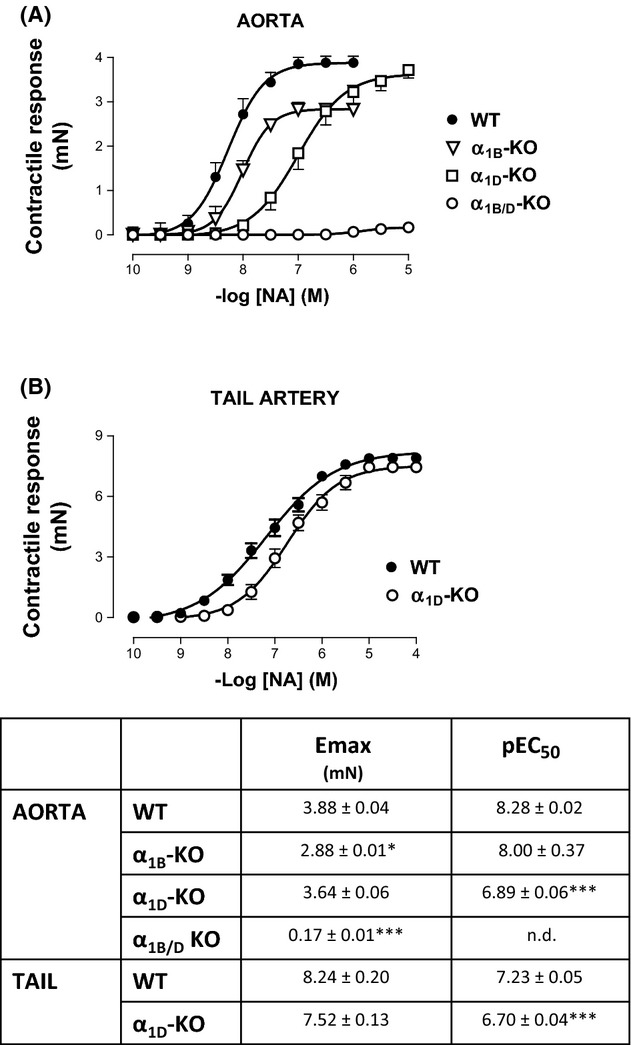
Contractile responses to cumulative concentrations of noradrenaline NA in aorta (A) or tail artery (B) of wild-type (WT), α_1D_-adrenoceptor knockout (α_1D_-KO), α_1B_-adrenoceptor knockout (α_1B_-KO), and α_1B/D_-adrenoceptor knockout (α_1B/D_-KO) mice. Emax (expressed as mN) and pEC_50_ of the concentration–response curves were included in each case. Values are represented as mean ± SEM of n = 3–6 experiments. Statistical significance was calculated by Student's *t* test. *P < 0.05, ***P < 0.001, n.d.= not determined.

CRC of NA were also performed in aortic and tail artery rings from α_1B_-KO, α_1D_-KO, α_1B/D_-KO, or WT mice and the results are shown in Figure 2. As in rat vessels, in WT mice the pEC_50_ of NA was significantly higher in aorta than in tail artery (8.28 ± 0.02 and 7.23 ± 0.05, respectively, *P* < 0.001). Comparing α_1D_-KO with WT, the pEC_50_ of NA was reduced in aorta and tail artery, although the maximal response (Emax) was not different (Fig. 2) in either case. No significant difference in potency of NA was observed between aortic rings from WT and α_1B_-KO mice although the Emax was significantly reduced. In α_1B/D_-KO, a contractile response of aorta was detectable only with the three highest concentrations of NA and was so small that the pEC_50_ of the CRC could not be calculated (Fig. 2A).

### NA-induced contraction exhibits a slower time-course in aorta than in tail artery

**Figure 3:**
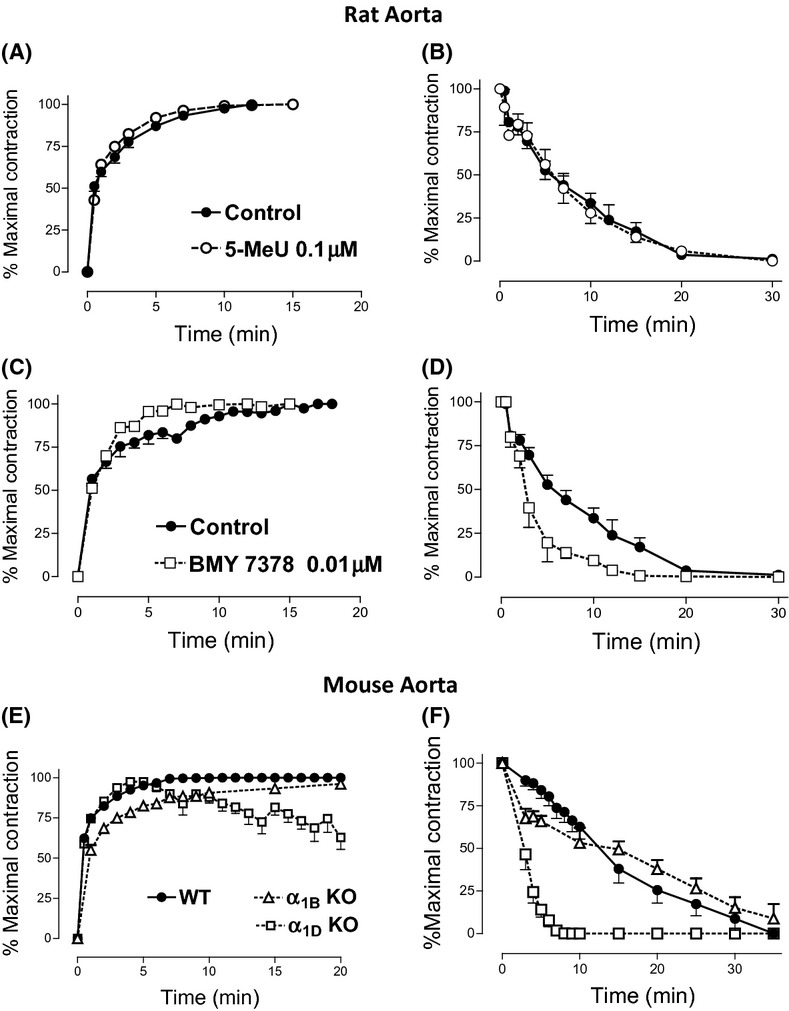
Time-course of the contractile response to noradrenaline in aorta from rat or transgenic mice. The magnitude of the contraction was determined at different times after addition of NA (1 μmol/L) to the bath chamber (A, C, and E) or after removal of the agonist (B, D, and F), in absence (control) or presence of selective antagonists (5-methylurapidil 0.1 μmol/L and BMY7378 0.01 μmol/L) or in different mouse strains: wild type (WT), α_1D_-adrenoceptor knockout (α_1D_-KO), α_1B_-adrenoceptor knockout (α_1B_-KO), and α_1B/D_-adrenoceptor knockout (α_1B/D_-KO). Values represent mean ± SEM of n = (3–6) experiments.

**Figure 4:**
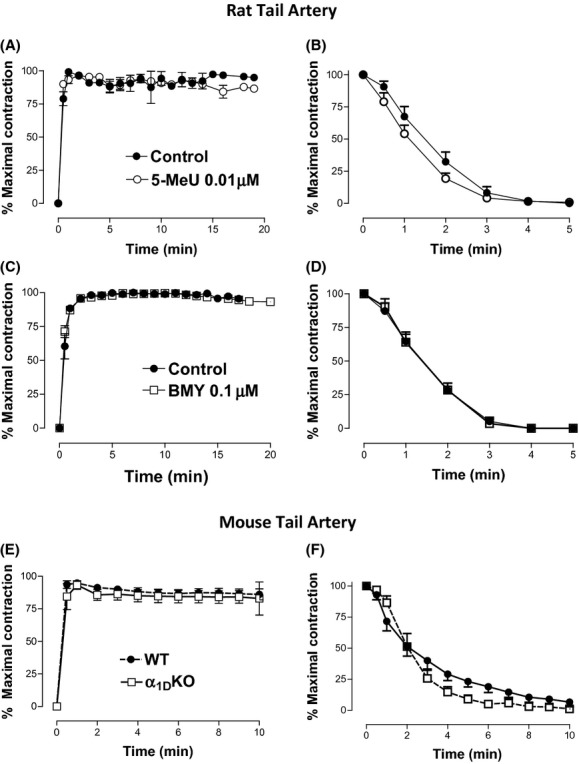
Time-course of the contractile response to NA in tail artery from rat or transgenic mice. The magnitude of the contraction was determined at different times after addition of NA (1 μmol/L) to the bath chamber (A, C and E) or after removal of the agonist (B, D, and F), in absence (control) or presence of selective antagonists (5-methylurapidil 0.01 μmol/L and BMY7378 0.1 μmol/L) or in different mouse strains: wild type (WT), α_1D_-adrenoceptor knockout (α_1D_-KO), α_1B_-adrenoceptor knockout (α_1B_-KO), and α_1B/D_-adrenoceptor knockout (α_1B/D_-KO). Values represent mean ± SEM of n = (3–6) experiments.

The concentration of NA needed to obtain the maximal response (1 μmol/L in aorta, 10 μmol/L in tail artery from rat and WT mice) evoked a contraction with different time-course profile in each vessel (Figs. 3 and 4).

In order to clarify the role that each α_1_-AR subtype plays in the distinctive time-course of the adrenoceptor-mediated response observed in each vessel, we analyzed changes in this response in the presence of subtype selective α1-antagonists in rat vessels or in knockout mice vessels (α_1B_-KO, α_1D_-KO, α_1B/D_-KO, or WT mice) with the objective of isolating the response to each receptor subtype.

We produced responses to single concentrations of NA (1 μmol/L in rat aorta and 10 μmol/L in tail artery) in the presence and absence of two selective antagonists, 5-methylurapidil, selective for α_1A_-ARs and BMY 7378 selective for α_1D_-AR (Koshimizu et al. 2002). The concentration of each antagonist was of the same order as its pA_2_ and/or pK_B_ in each vessel (Gisbert et al. 2003a).

In rat aorta, the kinetic of the NA-induced contraction was not affected by 5-methylurapidil (Fig. 3A), nor was the recovery of the basal tone after removal of the agonist (Fig. 3B). In the presence of BMY 7378 a slightly faster contractile response was observed (Fig. 3C) followed by a faster recovery of basal tone after washing the tissue. As Figure 3D shows, without antagonist, 5 minutes after agonist removal 50% of the maximal response to NA remained. However, in the presence of BMY, 5 minutes after NA removal the vascular tone was only 20% of the maximal contraction, and reached the basal levels around 10 minutes versus 20 minutes in the absence of BMY 7378.

In aorta from α_1D_-KO mice, the contractile response elicited by NA was faster than in WT and not sustained, with a slow decay after reaching its maximal response (Fig. 3E). In α_1B_-KO and α_1B/D_-KO mice, NA-induced contraction reached the maximal value more slowly than the contraction observed in aorta from WT (Fig. 3E). After removal of the agonist by washing, the return to the baseline was markedly slower in aorta from α_1B_-KO or WT mouse than in aorta from α_1D_-KO and α_1B/D_-KO mouse (Fig. 3F).

In rat tail artery, no significant changes were observed in the time-course profile of the NA-induced contraction in presence of any drug (Fig. 4A–D). In tail artery from the α_1D_-KO mouse the profile of the contractile response was similar to WT (Fig. 4E). The return to the baseline was only slightly slower in WT than in α_1D_-KO mouse (Fig. 4F) confirming the minor role of the α_1D_ subtype in this vessel.

### α_1_-adrenoceptors exhibit activity after removal of the agonist in aorta but not in tail artery

**Figure 5:**
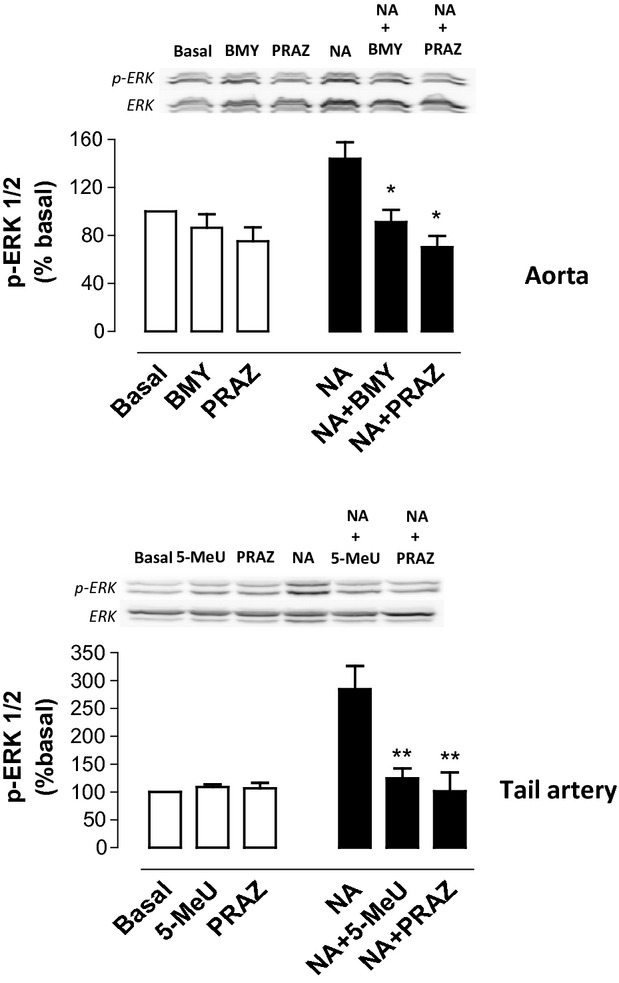
Basal and noradrenaline (NA)-induced phosphorylation of ERK1/2 in rat aorta and tail artery. When indicated, vessels were incubated or not with NA (10 μmol/L in aorta and tail artery) for 5 minutes, in absence or presence of selective ligands as prazosin (PRAZ), BMY 7378 (BMY), and 5-methylurapidil (5-MeU) at 1 μmol/L. After stimulation, cellular extracts were prepared as described under the methods section. Equal amounts (50 μg) of each sample were used to visualize the ERK1/2 expression (upper panels). The lower panels show equal amounts of ERK1/2 loaded on each sample. Bar graphics represents the quantification of basal (white bars) or NA-induced (black bars) ERK1/2 phosphorylation. Values represent means ± SEM of 3–4 independent experiments. Statistics was performed by the Dunnett's test *P < 0.05 versus NA.

Previous evidence indicates that cells expressing α_1D_-ARs exhibit elevated basal levels of calcium [32] and of pERK (McCune et al. 2000; Chalothorn et al. 2002) which could be decreased by α_1_-AR antagonists including the nonsubtype-selective prazosin or the α_1D_-AR-selective BMY 7378. In rat aorta, the basal level of pERK1/2 (in the absence of an adrenoceptor-mediated stimulus) was not significantly changed by BMY 7378 (1 μmol/L) or prazosin (1 μmol/L). No changes in basal phosphorylation of ERK1/2 were observed in rat tail artery incubated with 5-methylurapidil (1 μmol/L) or prazosin (1 μmol/L) (Fig. 5, white bars). As expected, NA induced an increase in the phosphorylation of ERK1/2 in both vessels, which was inhibited by prazosin and BMY 7378 in aorta and by prazosin and 5-methylurapidil in tail artery (Fig. 5, black bars).

**Figure 6:**
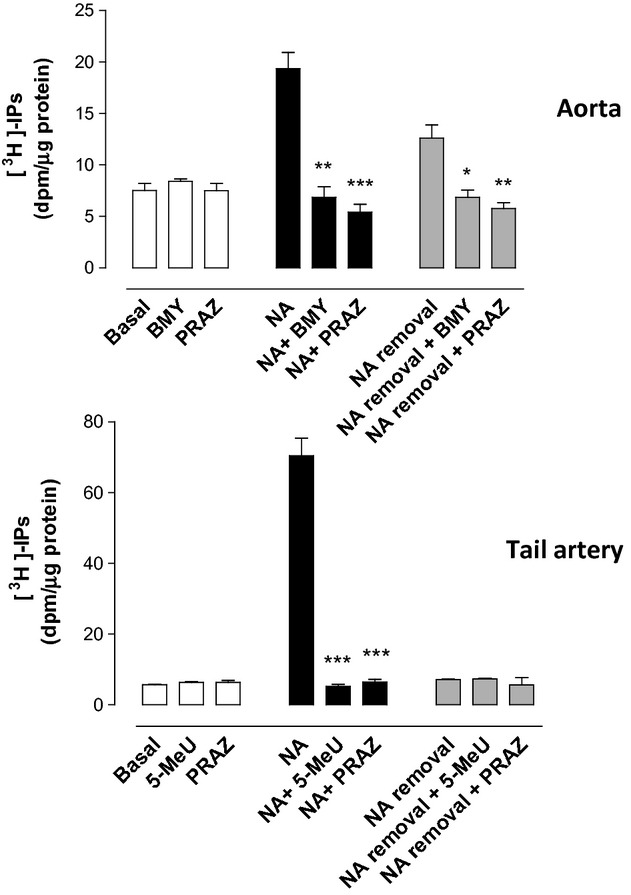
Inositol phosphates accumulation determined in rat aorta and tail artery in basal conditions (white bars), after addition of noradrenaline (NA) (black bars) and after addition and careful removal of NA (gray bars), according to the experimental procedure described in Methods. The experiments were performed in absence or presence of the selective ligands prazosin (PRAZ), BMY 7378 (1 μmol/L) (BMY), and 5-methylurapidil (5-MeU) at 1 μmol/L. Values represent means ± SEM of 3–4 independent experiments. Statistics was performed by the Dunnett's test *P < 0.05, **P < 0.01, ***P < 0.001 versus noradrenaline or noradrenaline removal. NA, noradrenaline 1 μmol/L in aorta, and 10 μmol/L in tail artery.

The incubation with selective antagonists did not change the basal IPs levels in rat aorta, nor in rat tail artery (Fig. 6, white bars). Addition of NA (1 μmol/L in aorta, 10 μmol/L in tail artery) induced a marked increase in the IPs accumulation which was completely inhibited by prazosin (1 μmol/L) and by BMY 7378 (1 μmol/L) or 5-methylurapidil (1 μmol/L) in aorta or tail artery, respectively (Fig. 6, black bars).

Following the experimental procedure described in the methods section, we had previously observed in aorta but not in tail artery a population of active α_1D_-ARs that increases IPs accumulation after removal of the agonist (Gisbert et al. 2003b). The same results were obtained in the present work. Figure 6 (gray bars) quantifies the magnitude of the IPs accumulation in aorta observed after agonist removal, and shows that incubation with prazosin (1 μmol/L) or BMY 7378 (1 μmol/L) inhibits it. Different results were observed in tail artery. No increase in IPs was observed after removal of the agonist and incubation with prazosin (1 μmol/L) or 5-methylurapidil (1 μmol/L) did not modify IPs levels (Fig. 6, gray bars).

**Figure 7:**
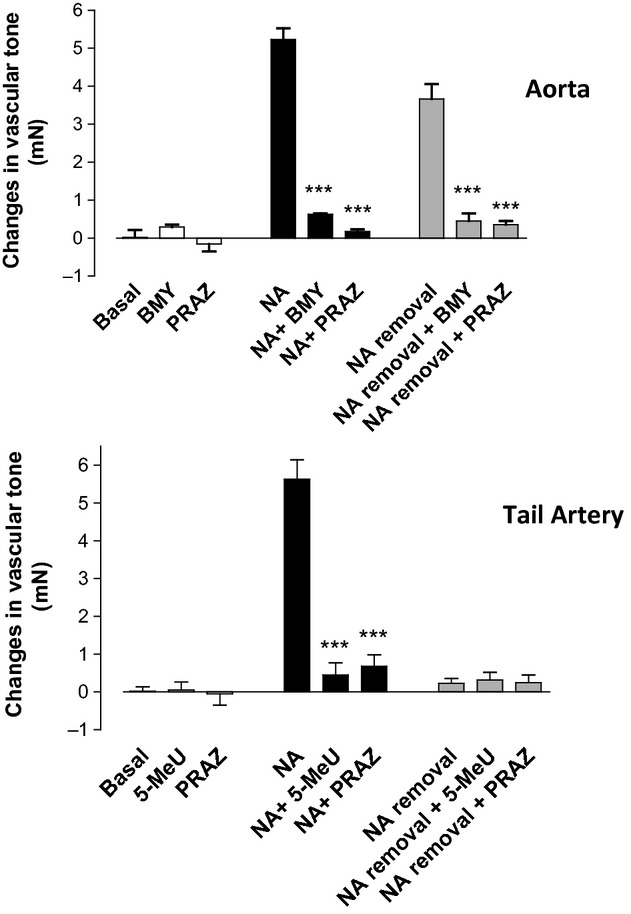
Changes in the vascular tone observed in rat aorta and tail artery in basal conditions (white bars), after addition of noradrenaline (black bars) and after addition and careful removal of noradrenaline (gray bars) according to the experimental procedure described in the methods section. The experiments were performed in absence or presence of the selective ligands prazosin (PRAZ), BMY 7378 (BMY), and 5-methylurapidil (5-MeU) at 1 μmol/L. Values represent means ± SEM of 4–5 independent experiments. Statistical analysis was performed by the Dunnett's test: ***P<0.001 to test the effects of antagonists versus noradrenaline or noradrenaline removal. NA, noradrenaline 1 μmol/L in aorta and 10 μmol/L in tail artery.

None of the antagonists assayed modified the basal tone of the rat aorta or tail artery (Fig. 7, white bars) and, as expected, addition of NA to the bath chamber promoted a sustained increase in tone that was almost completely inhibited in presence of prazosin or either BMY 7378 or 5-methylurapidil (Fig. 7, black bars). After careful removal of the agonist and following the experimental procedure previously described, a spontaneous increase in the vascular tone was observed in aorta but not in tail artery (Fig. 7, gray bars). Incubation with prazosin (1 μmol/L) or BMY 7378 (1 μmol/L) inhibits this increase in tone observed after agonist removal, as has been previously shown (Gisbert et al. 2000, 2003b).

**Figure 8:**
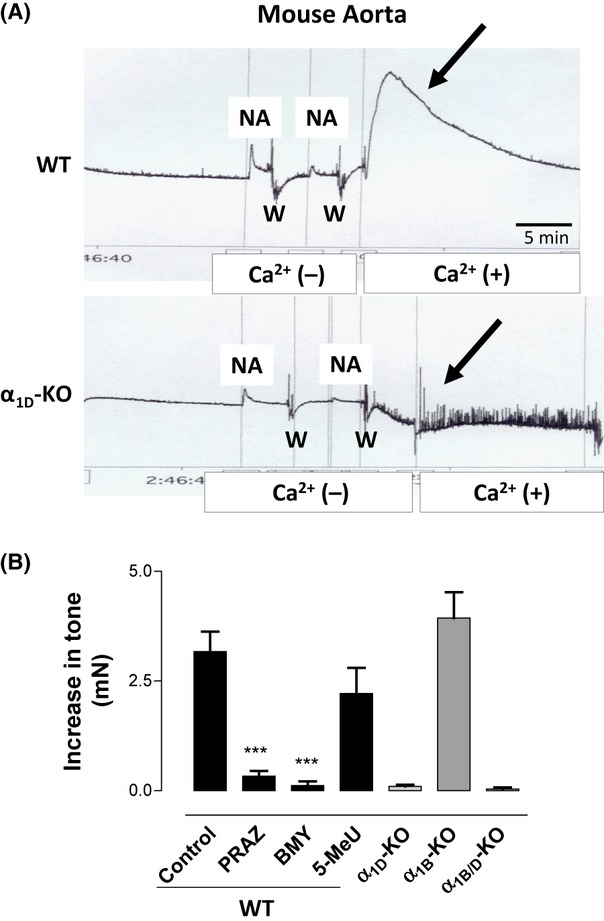
(A) Representative tracings of the changes in tone observed in aorta of wild-type (WT) and α_1D_-knockout (α_1D_-KO) mice after addition of NA (1 μmol/L) in a calcium-free medium, subsequent removal of the agonist and washing (W), and subsequent incubation in a calcium-containing solution. Arrows show the spontaneous increase in tone observed in aorta from WT but not from α_1D_-KO mouse. (B) Quantification of the spontaneous increase in tone observed in aorta from WT mice incubated with or without the selective ligands prazosin (PRAZ), BMY 7378 (BMY), and 5-methylurapidil (5-MeU) at 1 μmol/L (black bars), and in α_1B_-KO, α_1D_-KO, and α_1B/D_-KO mice (gray bars). Values represent means ± SEM of 4–5 independent experiments. Statistics was performed by the Dunnett's test, ***P < 0.001 to test the effect of antagonists versus WT

In order to elucidate if an inadequate washing or tissue peculiarity rather than a special activity of one α_1_-AR subtype could explain the increase in tone observed after NA removal, we performed the same experimental procedure in vessels from knockout mice. Experiments in aorta from WT and α_1B_-KO mice show a spontaneous increase in tone after NA-removal similar to that found in rat aorta. However, a similar increase in tone was not observed in aorta from α_1D_-KO or α_1D_/_1B_-KO mice after NA-removal (Fig. 8). The spontaneous increase in tone observed in WT mouse was completely inhibited by prazosin (1 μmol/L) and BMY 7378 (1 μmol/L), but not by 5-methylurapidil (1 μmol/L) (Fig. 8B).

## Discussion and Conclusions

The major findings of the present study are that the α_1D_-subtype is responsible of the greater contractile sensitivity, slower time-course and postactivation contraction to an adrenoceptor-mediated stimulus in conductance vessels. This conclusion can be drawn from differences observed in the contractile response to NA between a conductance artery (aorta) and distributing artery (tail artery) in two species, the receptor subtype being isolated pharmacologically in the rat and by receptor subtype knockout in the mouse.

### Conducting arteries respond to adrenoceptor-mediated stimulus with higher sensitivity than distributing vessels

Rat aorta exhibited a higher sensitivity for NA than tail artery and this difference was observed independently of the signaling pathway analyzed: IPs accumulation, ERK1/2 phosphorylation, or contractile response. Interestingly when a measure of maximal response representing efficacy was calculated this was not greater in aorta so a generally “larger” response signal per se is not implicated.

It is well known that rat, mouse, and human aorta express protein of the three α_1_-ARs subtypes but, in functional terms, the vasoconstrictor role of α_1D_ is predominant (Kenny et al. 1995; Hussain and Marshall 1997; Gisbert et al. 2000, 2002, 2003a; Yamamoto and Koike 2001; Hosoda et al. 2005), whereas in rat tail artery the α_1A_-subtype is mainly implicated in adrenoceptor-mediated contraction (Gisbert et al. 2003a; Martí et al. 2005; Docherty 2010). Thus, the greater sensitivity to NA observed in aorta could be attributed to the main functional role played by α_1D_-AR in this vessel. This proposal was based on previous reports showing a higher potency of NA and adrenaline on cloned α_1D_-ARs expressed in different cell lines (Theroux et al. 1996; Pérez-Aso et al. 2013), as well as aorta and other conducting arteries where the α_1D_ subtype plays a main functional role (Daly et al. 2002; Tanoue et al. 2002; Deighan et al. 2005; Hosoda et al. 2005; Methven et al. 2009a,b).

However, in native tissues, changes in potency of the agonists could be also explained by structural or cellular characteristics of vessels independent of the α_1_ subtype involved. The present results obtained with gene-targeted mice confirmed the higher sensitivity of the native α_1D_ subtype to NA as responsible for the higher potency exhibited by the agonist in aorta since, in the mouse model lacking the α_1D_-AR (α_1D_-KO), the pEC_50_ of NA was significantly lower than that observed in aorta from α_1B_-KO or WT mice; α_1B/D_-KO compared with α_1B_-KO gave a similar result. Moreover, the much smaller difference in potency and efficacy of CRC to NA observed in tail artery between α_1D_-KO and WT mice suggests a lesser role for α_1D_-ARs in this vessel; an earlier study using α_1B_-KO showed also a lesser role for α_1B_-AR in this artery (Daly et al. 2002).

### After removal of the adrenoceptor-mediated stimulus, the contractile response disappears more slowly in conducting than in distributing arteries

Aorta from rat or mouse exhibits a distinctive time-course in the contractile response to an adrenoceptor-mediated stimulus. The time-course profile of this response is characterized by a slow decay in the contractile tone when the agonist was removed. We have previously described a similar time-course in other conducting vessels such as iliac and proximal mesenteric arteries, where α_1D_-AR plays a functional role (Ziani et al. 2002).

On the contrary, in distributing vessels such as tail artery, or resistance vessels such as small mesenteric branches, where the α_1D_-AR has not a predominant role, and the α_1A_-AR is the main subtype involved, this slow time-course is not observed, and a fast decay in the contractile tone after agonist removal was observed (Ziani et al. 2002). Therefore, we can attribute the slower time-course profile to the presence of the α_1D_-subtype in a vessel but also it could be due to structural differences between arteries. The use of selective antagonists in rat aorta as well as studies in vessels from mice lacking the α_1D_-subtype confirms the involvement of this receptor in the distinctive time-course observed in conducting vessels.

A selective antagonist of the α_1D_-ARs, BMY 7378, but not the α_1A_ selective antagonist 5-methylurapidil (Michelotti et al. 2000; Koshimizu et al. 2002), affects to a great extent the recovery of basal tone after agonist removal. This difference was even more evident in knockout mice. In strains where the α_1D_-AR was not expressed (α_1D_-KO and α_1B/D_-KO), the recovery of basal tone was almost complete 5 minutes after agonist washing whereas it takes up 30 minutes in WT and α_1B_-KO mice. In tail artery from all strains, the decay in this maximal response to NA was faster than in aorta from rats or WT mice, and similar to aorta from α_1D_-KO and α_1B/D_-KO mouse.

In conclusion, after removal of the agonist, a faster decay in the contractile tone was observed in aorta from α_1D_-KO and α_1B/D_-KO mice versus WT and α_1B_-KO, and this time-course profile of the adrenoceptor-mediated contraction is similar to that observed in distributing vessels such as tail artery. Therefore, a consequence of α_1D_-ARs activation in conducting vessels is a sustained contractile response when the stimulus disappears.

The next question that arises was the possible involvement of the constitutive activity of α_1D_-ARs in this sustained response. It has been reported that cloned α_1D_-ARs exhibit constitutive activity, evidenced by increased levels of calcium (García-Sainz and Torres-Padilla 1999a) or pERK1/2 (McCune et al. 2000) that were selectively inhibited by prazosin or BMY 7378, acting as inverse agonists.

After incubation with prazosin or BMY 7378, no change was registered in p-ERK1/2 IPs or contractile tone in aorta, which suggests that constitutive activity observed in cloned α_1D_-ARs is not so evident in native receptors and has not a relevant impact on the signaling pathway or in the vascular tone. Therefore, there is no evidence for the presence of a population of constitutively active α_1D_-AR, coupled to p-ERK signal with a modulator role on the basal vascular tone in aorta.

Interestingly, after activation by NA and removal of the agonist, the α_1D_-ARs continue actively coupled to the IP pathway and, at the same time, we observed a temporary increase in vascular tone when NA was no longer present in the bath. The experiments performed in knockout mice confirmed this peculiarity of α_1D_-ARs as the increased tone which appears after agonist removal was observed only in WT and α_1B_-KO mouse, but not in α_1D_-KO or α_1B/D_-KO mice.

Recent evidences indicate that α_1D_-ARs are expressed as a multiprotein complex at the plasma membrane (Lyssand et al. 2008, 2010) interacting with the syntrophin family through a PSD95/DlgA/Zo-1 (PDZ)-domain (Chen et al. 2006). Syntrophin isoforms play selective roles in the α_1D_-AR/dystrophin-associated protein complex signalosome as α-syntrophin increases α_1D_-AR binding site density while β_2_-syntrophin enhances α_1D_-AR coupling to downstream signaling effectors (Lyssand et al. 2011). In addition, this signaling complex is not mimicked by the α_1A_ or α_1B_-AR subtypes which suggest that it could be involved in the peculiar activity exhibited by the α_1D_ subtype.

The characteristic behavior of the α_1D_-AR has been previously reported (Gisbert et al. 2000, 2003b; Ziani et al. 2002) as constitutive activity which manifests only after agonist stimulation and removal. Thus, in native vascular smooth muscle, the α_1D_-ARs remain constitutively active after agonist activation, and maintain the adrenoceptor-mediated response when the agonist is removed; finally, α_1D_-ARs are internalized and the vessel recovers the basal tone. However, this activity observed after removal of the agonist could be also attributed to a prolonged binding of NA to α_1D_-ARs which activates them for a while. As we have discussed in previous papers, this explanation does not hold (Gisbert et al. 2000, 2003b) but in any case, the more interesting result is not related to the fact that the activity showed by α_1D_-ARs after removal of the agonist was “truly” or only “apparent” constitutive activity. The more interesting question is the physiological consequence of this activity which explains the slower decay observed in the adrenoceptor-mediated response when the agonist is removed.

In fact, aortas from α_1D_-KO and α_1B/D_-KO mice, which did not exhibit a spontaneous increase in tone after agonist removal, had a time-course profile of recovery of the basal tone faster than aorta from WT and α_1B_-KO mouse, and similar to tail artery from any strain.

### Physiological and therapeutic relevance of α_1D_-ARs in conductance vessels

The present results show that, in response to a systemic adrenoceptor-mediated stimulus, a poorly innervated conductance vessel such as aorta, where α_1D_-AR is the main functionally relevant subtype, responds with higher sensitivity than a vessel where the α_1A_-AR subtype is dominant, as occurs in tail artery. Thus, plasma levels of catecholamines, which rarely exceed 10 nmol/L (Goldstein et al. 2003) could induce a moderate adrenoceptor-mediated response in conductance vessels. This contractile response can be temporarily sustained when the agonist is removed, due to the constitutive coupling of the α_1D_-subtype to IPs/contraction pathway after agonist removal, and this mechanism would prevent abrupt changes in the caliber of conductance arteries when the adrenoceptor-mediated stimulus fluctuates.

However, the higher threshold for α_1A_-adrenoceptors present in tail artery might take them out of the reach of circulating levels of catecholamines. Thus the α_1A_-ARs might require the high local concentrations produced only by release of noradrenaline from perivascular nerves. In this case, the response is fast and intense, and disappears when the stimulus does. This mechanism would permit the fine adjustment of the contractile tone of distributing vessels by the local nervous stimulus and consequently the precise adjustment of blood flow. This concept is consistent with the hypothesis of Stassen et al. (1998) that α_1A_-adrenoceptors are present in blood vessels only when adrenergic nerves are present and might add the further idea that α_1A_-ARs are activated physiologically only or mainly by nerves (Daly et al. 2002).

The differential role exhibited by the α_1D_ and the α_1A_ subtypes, present in conductance or distributing and resistance vessels, respectively, opens new lines of pharmacological research looking for the selective modulation of a given subtype as a more vessel selective, accurate, and safe strategy to control vascular tone.

**Figure 9:**
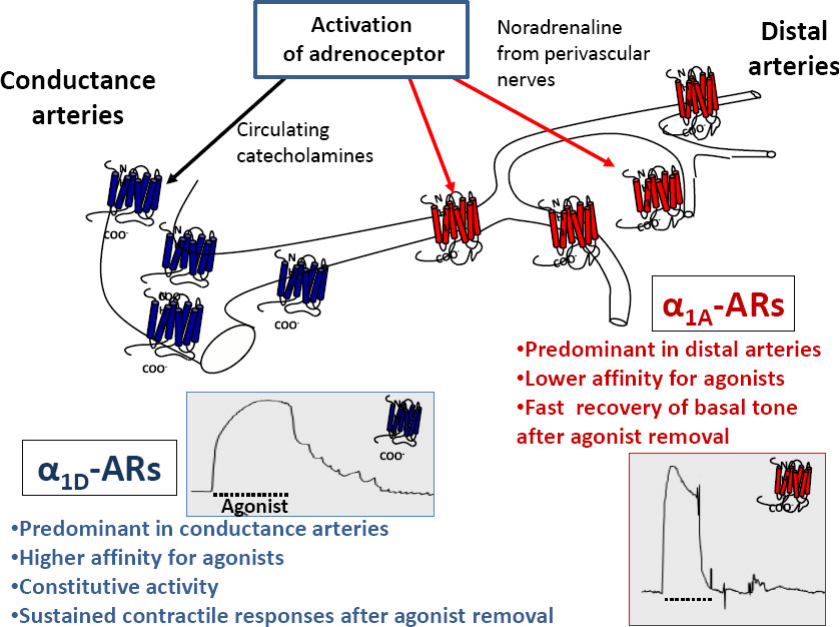
Schematic picture showing that preponderance of the most sensitive α_1D_-AR subtype in noninnervated conductance arteries such as aorta, allows responsiveness to physiological levels of circulating catecholamines. The activity showed by this subtype after agonist removal sustains the contractile tone and prevents abrupt changes in vessel caliber when the stimulus fluctuates. In innervated distributing arteries, high local concentrations of NA are required to activate the less sensitive subtype of α_1A_-adrenoceptors which elicit a response that is rapid but short lived, allowing fine adjustment of the contractile tone by perivascular sympathetic nerves.

In conclusion, as Figure 9 depicts, high sensitivity to agonist and persistence of response after agonist removal is a property of α_1D_-adrenoceptors. Therefore, the preponderance of this subtype in noninnervated conductance arteries such as aorta allows responsiveness to circulating catecholamines and prevents abrupt changes in vessel caliber when the stimulus fluctuates. Conversely, in innervated distributing arteries, high local concentrations of NA are required to activate α_1A_-adrenoceptors for a response that is rapid but short lived allowing fine adjustment of the contractile tone by perivascular sympathetic nerves.

## Abbreviations

AR: α_1_-adrenoceptors
CRC: Concentration–response curves
EDTA: ethylenediaminetetraacetic acid
IP: inositol phosphate
KO: knockout
MAPK: mitogen-activated protein kinase
NA: noradrenaline
PBS: phosphate-buffered saline
PDZ: PSD95/DlgA/Zo-1
PVDF: polyvinyldiene fluoride
WT: wild-type
